# Differential sensitivity of acute myeloid leukemia cells to daunorubicin depends on P2X7A versus P2X7B receptor expression

**DOI:** 10.1038/s41419-020-03058-9

**Published:** 2020-10-18

**Authors:** Anna Pegoraro, Elisa Orioli, Elena De Marchi, Valentina Salvestrini, Asia Milani, Francesco Di Virgilio, Antonio Curti, Elena Adinolfi

**Affiliations:** 1 Department of Medical Sciences, University of Ferrara, 44121 Ferrara, Italy; 2 Department of Haematology and Oncology, University Hospital S.Orsola-Malpighi, Institute of Haematology “L. and A. Seràgnoli”, 40138 Bologna, Italy

**Keywords:** Haematological diseases, Preclinical research

## Abstract

Acute myeloid leukemia (AML) is a common adult leukemia often arising from a preexistent myelodysplastic syndrome (MDS). High mortality rates of AML are caused by relapse and chemoresistance; therefore, we analyzed the role of P2X7 receptor (P2X7R) splice variants A and B in AML progression and response to chemotherapy. The expression of P2X7RA and P2X7RB was investigated in samples obtained from MDS and AML untreated subjects or AML patients in relapse or remission after chemotherapy. Both P2X7RA and P2X7RB were overexpressed in AML versus MDS suggesting a disease-promoting function. However, in relapsing patients, P2X7RA was downmodulated, while P2X7RB was upmodulated. Treatment with daunorubicin (DNR), one of the main chemotherapeutics for AML, upregulated P2X7RB expression while reducing P2X7RA mRNA in AML blasts. Interestingly, DNR administration also caused ATP release from AML blasts suggesting that, following chemotherapy, activation of the receptor isoforms via their agonist will be responsible for the differential survival of blasts overexpressing P2X7RA versus P2X7RB. Indeed, AML blasts expressing high levels of P2X7RA were more prone to cell death if exposed to DNR, while those overexpressing P2X7RB were more vital and even protected against DNR toxicity. These data were reproducible also in HEK-293 cells separately expressing P2X7RA and B. P2X7RA facilitation of DNR toxicity was in part due to increased uptake of the drug inside the cell that was lost upon P2X7RB expression. Finally, in an AML xenograft model administration of DNR or the P2X7R antagonist, AZ10606120 significantly reduced leukemic growth and coadministration of the drugs proved more efficacious than single treatment as it reduced both P2X7RA and P2X7RB levels and downmodulated c-myc oncogene. Taken together, our data suggest P2X7RA and P2X7RB as potential prognostic markers for AML and P2X7RB as a therapeutic target to overcome chemoresistance in AML relapsing patients.

## Introduction

Acute myeloid leukemia (AML) is a clonal disorder characterized by the proliferation and the accumulation of myeloid precursors in the bone marrow (BM) that show aberrant differentiation patterns leading to hematopoietic impairment^1^. In some cases, AML can originate from an evolving MDS, i.e., a clonal disorder of hematopoietic stem cells^2^. Despite several efforts have been made to improve the clinical outcome of AML, current drugs fail to eliminate the leukemic stem cells responsible for the disease^1^. Therefore, the prognosis remains poor with most AML patients developing drug resistance. Accordingly, the identification of new progression/relapse biomarkers and potential therapeutic targets remains an urgent clinical goal in AML^3^.

The P2X7 receptor (P2X7R) emerged as a promising therapeutic target in oncology, due to its widespread expression in cancer cells and the efficacy of its antagonist in reducing cancer growth and dissemination in animal models^4–6^. Haemopoietic lymphoproliferative disorders, among which AML, were the first neoplasias where P2X7R oncogenic activity was demonstrated^7–9^. However, evidence on the role of ATP and P2X7R in leukemia etiopathogenesis remains limited^10–12^.

P2X7R is an ATP-gated ion channel that, when stimulated with high concentrations of agonists, triggers cell death via the opening of a large nonselective plasma membrane pore (macropore)^13–15^. Formation of the macropore is dependent upon the presence of the long intracellular carboxyl tail as its deletion prevents pore formation while leaving unaltered the ion channel activity^14,16^. P2X7R activated macropore opening was also shown to enhance the intracellular uptake of drugs including chemotherapeutics such as doxorubicin^17^ and was therefore proposed as tumor cell-specific drug delivery system^18,19^. The structure of P2X7R C terminal was recently reported^20^ and it was speculated that this region was acquired by genomic rearrangement from a P2X4R like-gene in ancient jawed vertebrates generating the actual mammalian P2X7R^21^. It is therefore not surprising that certain species such as humans still express functional P2X7R splice variants missing the C terminal domain such as P2X7RB, an isoform unable to form the macropore, and thus without cytotoxic activity, but still endowed with ion channel properties^22,23^. In recent years, others and we demonstrated a growth-promoting activity for P2X7RB in cell lines^23,24^ and osteosarcoma^24^ as well as its involvement in metastasis and transformation^25,26^. The tumor-promoting activity of P2X7RB is shared by the full-length P2X7RA isoform accelerates cancer growth and dissemination and associates with poor prognosis in different malignancies^5,27–29^. However, to our knowledge, the effect of in vivo P2X7R blockade or chemotherapy on P2X7R isoforms expression was never tested and an extensive analysis of the distinct behavior of P2X7RA and P2X7RB in AML patients was never performed. The present study was aimed at identifying P2X7RA and B as novel biomarkers of AML response to chemotherapy and as new therapeutic targets for the disease. We covered P2X7R isoforms expression in an MDS and AML population and their behavior in vitro and in vivo models of the pathology, following chemotherapy and P2X7R antagonist administration.

## Materials, subjects, and methods

### Reagents

RPMI 1640, Iscove’s Modified Dulbecco’s Medium, nonessential amino acids, hygromycin, daunorubicin (DNR), and BzATP were from Sigma-Aldrich (Milan, Italy). DMEM high glucose medium, fetal bovine serum, penicillin, and streptomycin solutions were acquired from Euroclone (Milan, Italy). AZ10606120 was purchased from Tocris Bioscience (Bio-Techne, Bristol, UK). The reverse transcription kit, Real-Time PCR TaqMan reagents, Halt^TM^ Protease and Phosphatase Inhibitor Cocktail, EDTA-Free were acquired from Thermo Scientific.

### Clinical samples

Primary leukemic cells were used for mRNA extraction and obtained, as previously described^10^, from the peripheral blood or BM of 68 patients subdivided according to diagnostic phase in 10 myelodysplastic syndrome (MDS), 47 first diagnosed of AML, 6 with relapse of the disease after chemotherapy, and 5 remitting AML patients. The main patients’ clinical characteristics are summarized in Supplementary Table 1. All patients samples were obtained upon signed informed consent, our research was approved by the Ethics Committee of Policlinico S. Orsola-Malpighi, University Hospital of Bologna (approval code: 94/2016/O/Tess).

### Real-time quantitative RT-PCR

Total RNA was extracted using the PureLink RNA Mini Kit (Thermo Scientific). Real-time quantitative PCR was performed as previously described^23^. A comparative CT experiment (ΔΔCT) was run to allow determination of fold increase of the target cDNA in the test sample relative to Te85 cell line reference sample, which was previously demonstrated to express minimal levels of both P2X7RA and B^24^. Customized TaqMan (Thermo Scientific, Applied Biosystems) primers and probes were for P2X7RA: forward primer: 5′ CGGCTCAACCCTCTCCTACT‐3′; reverse primer: 5′GGAGTAAGTGTCGATGAGGAAGTC 3′; 3′FAM probe: 5′ CACAGCGGCCAGACCG 3′. For P2X7RB, forward primer: 5′ GGAAAATGGTTTGGAGAAGGAAGTG 3′; reverse primer: 5′ CGATGAGGAAGTCGATGAACACA 3′; 3′FAM probe: 5′ ACAAGCGCTGCGTTAGT 3′. GAPDH reference was a Pre-Developed TaqMan Assay (Hs02786624_g1, Thermo Scientific, Applied Biosystems).

### Cells

HL-60 human promyelocytic, HEK-293 stably expressing P2X7RA, P2X7RB, or empty vectors (MOCK) cell lines were previously available at Adinolfi laboratory^11,12,23^ and periodically tested for the absence of mycoplasma by MycoAlert kit (Euroclone). Culture media were RPMI 1640 medium plus nonessential amino acids (HL-60 and AML blasts) or DMEM high glucose (HEK-293), supplemented with 10% fetal bovine serum, 100 U/ml penicillin and 100 mg/ml streptomycin.

### Immunoblots

Cell lysates or tumor homogenates were loaded in 4–12% NuPAGE Bis-Tris precast gels (Thermo Scientific) and proteins were separated and transferred onto a nitrocellulose blotting membrane (Amersham Protran, GE Healthcare, USA). Membranes were incubated overnight at 4 °C with primary antibodies as follows: anti-P2X7 (P8232, Sigma-Aldrich) antibody, recognizing the C terminal domain of the protein was diluted 1:300, anti-P2X7 (APR008, Alomone Labs, Jerusalem, Israel) antibody, recognizing the extracellular loop of the protein was diluted 1:300, anti-c-myc antibody (A190-105A, Bethyl Laboratories, INC) was diluted 1:2500, and anti-SKP-1 antibody (MA5-15928, Thermo Scientific) was diluted 1:1000. Membranes were then incubated with secondary goat anti-rabbit (170-6515, BIORAD) or goat anti-mouse (170-6516, BIORAD), HRP-conjugated antibodies at a 1:3000 dilution. When required blocking peptide for the APR008 antibody (BLP-PR008, Alomone Labs) was added to the primary antibody at a 1:2 ratio and preincubated for 5 h before proceeding with the above-described immunostaining protocol. Protein bands were visualized by ECL HRP Chemiluminescent Substrate ETA C ULTRA 2.0 (Cyanagen Srl, Bologna, Italy) with a Licor C-Digit Model 3600. Densitometric analysis was carried out with ImageJ software and data were normalized on SKP-1 content.

### P2X7R activity assays

Measurement of intracellular calcium concentration with FURA-2AM and of plasma membrane permeabilization to ethidium bromide was performed as previously described^30,31^.

### ATP measurement

ATP concentration was evaluated with ENLITEN rLuciferase/Luciferin reagent (Promega, Milan, Italy), according to the manufacturer’s instructions as previously described^12^.

### Cells viability assays

AML blasts were resuspended in RPMI medium and treated with PBS or 200 nM DNR for 6 h. HEK cells seeded in serum-free medium let adhere and subsequently treated with PBS or 200 nM DNR for 48 h. Cell numbers were assessed at time 0 and 6 (blasts) or at time 0, 24, and 48 h (HEK). The Alamar blue assay was performed as per the manufacturer’s instructions after 24 h of incubation with either PBS or 200 nM DNR.

### DNR permeabilization assays

HEK MOCK, P2X7RA, and P2X7RB were imaged with a Nikon Eclipse TE300 inverted microscope (Nikon, Japan), while HIGH-P2X7RA and HIGH-P2X7RB AML blasts with a Leica DMI 4000B (Leica Microsystems CMS GmbH) microscope in saline solution (125 mM NaCl, 5 mM KCl, 1 mM MgSO_4_, 1 mM NaH_2_PO_4_, 20 mM HEPES, 5.5 mM glucose, 5 mM NaHCO_3_, and 1 mM CaCl_2_, pH 7.4) plus 1 µg/ml DNR and whenever required 3 mM ATP. Fluorescence was captured at 328 nm excitation and 545 nm emission.

### In vivo experiments

Animal procedures were approved by the University of Ferrara ethic committee and the Italian Ministry of Health. In vivo experiments were performed in the athymic nude-Foxn1nu strain acquired from Envigo (San Pietro al Natisone, Italy). A sample size of 12 animals per condition was computed a priori with the G*power software^32^ based on previous data obtained with AZ10606120^33^ and assuming an effect size of 1 and a power of 85%. Four-to-six weeks old female mice were subcutaneously injected with 5 × 10^6^ HL-60. Animals were randomized with the randomizer software (www.randomizer.org) to receive intramass injections of sterile PBS (placebo), DNR (150 µg/ml), AZ10606120 (2 μM), or both compounds at post-inoculum days 8 and 10 as previously described^33^. The operator was blinded to the group of allocation. Tumor size was measured at post-inoculum days 8, 10, and 12 and ex vivo with a caliper and volume calculated according to the following equation: volume = π/6[w1 × (w2)^2^], where w1 = major diameter and w2 = minor diameter. Mice were euthanized 12 days after cell inoculum and excised tumors were homogenized and processed for further analysis.

### Statistics

**Fig. 1:**
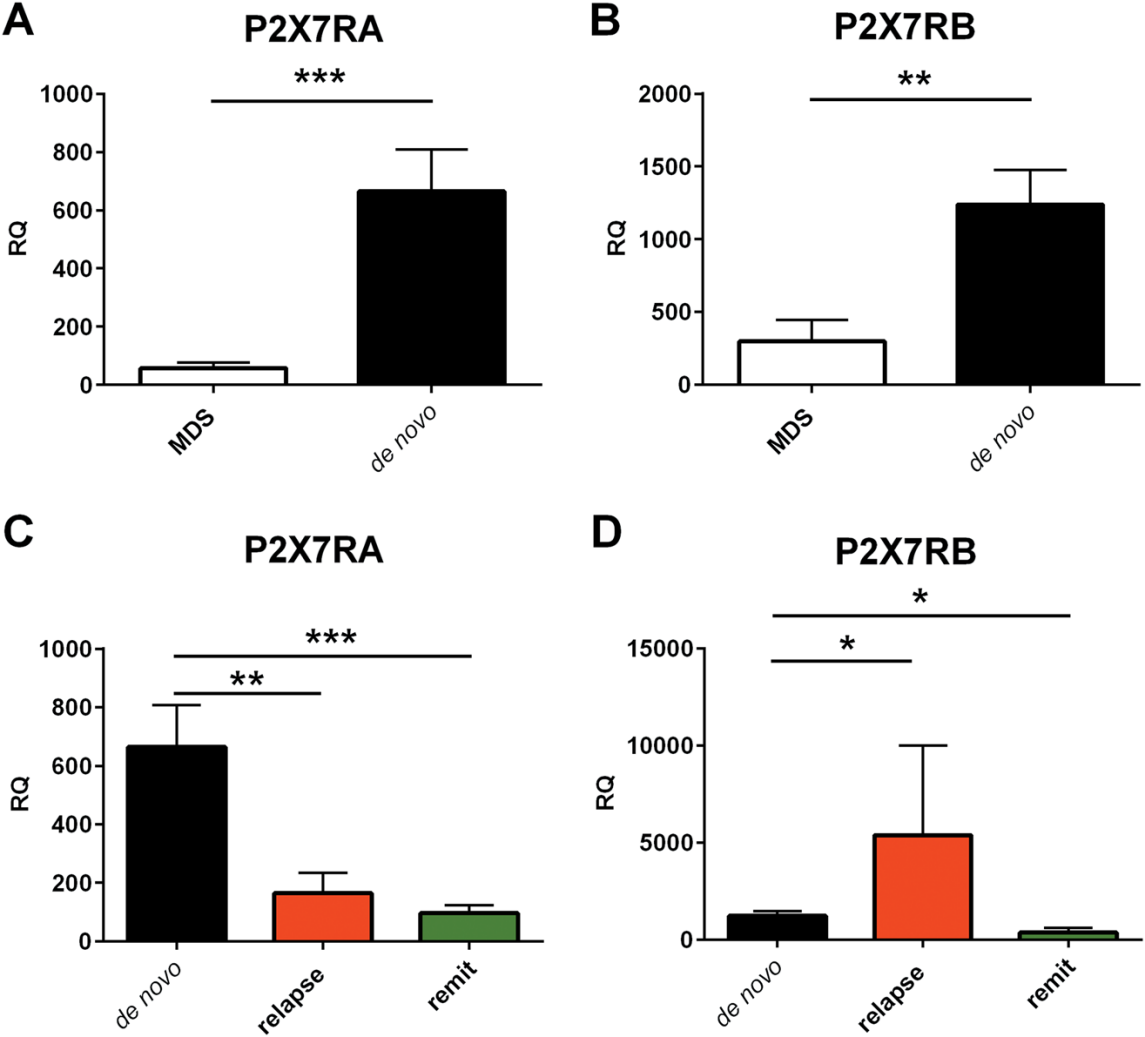
P2X7R isoforms A and B expression in MDS and AML patients. **a**, **b** Both P2X7RA and P2X7RB isoforms mRNA are overexpressed in de novo AML as compared to MDS patients. **a** mRNA was extracted from primary leukemic cells from MDS and de novo AML patients and expression of P2X7R isoform A was compared (MDS, white, *n* = 10, de novo AML, black, *n* = 47). **b** mRNA was extracted from primary leukemic cells from MDS and de novo AML patients and expression of P2X7R isoform B was compared (MDS, white, *n* = 10, de novo AML, black, *n* = 47). **c**, **d** Within the AML group P2X7R isoforms are differently expressed in relapsing patients as compared to remitting patients. **c** Expression of P2X7R isoform A within the AML patient’s group by subdividing into de novo (black), relapsing (red), remitting (green) (de novo *n* = 47, relapsing *n* = 6, remitting *n* = 5). **d** Expression of P2X7R isoform B within the AML patient’s group by subdividing into de novo (black), relapsing (red) and remitting (green). (de novo *n* = 47, relapsing *n* = 6, remitting *n* = 5). Data are represented as mean ± SEM. *P ≤ 0.05; **P ≤ 0.01; ***P ≤ 0.001.

All data are shown as mean ± standard error of the mean (SEM). Except for real-time PCR data reported in Fig. 1, where Welch’s correction was applied due to different sample sizes, significance was calculated assuming equal standard deviations and variance, with a two-tailed Student’s *t* test performed with the GraphPad Prism software (La Jolla, CA, USA). Coding: *P ≤ 0.05; **P ≤ 0.01; ***P ≤ 0.001; ****P ≤ 0.0001.

## Results

### P2X7RA and P2X7RB isoforms are overexpressed in newly diagnosed AML compared to MDS and differentially expressed in AML relapsing patients

P2X7R expression in AML was already reported in the past^10^, however the analysis of the differential expression of P2X7RA and B isoforms in the disease was missing. Therefore, we investigated by Real time PCR mRNA levels of P2X7RA and B in 58 patients affected with AML and 10 patients affected with MDS. AML patients were further subdivided according to the diagnostic phase and response to treatment into three groups: (1) newly diagnosed untreated subjects (de novo, *n* = 47), (2) relapsing subjects, with a return of the pathology after chemotherapy (relapsing, *n* = 6), or (3) remitting patients, with no evident reappearance of the pathology one to three months after chemotherapy (remitting, *n* = 5). mRNA levels of P2X7RA and B of AML de novo patients were massively increased as compared to MDS blasts, supporting the hypothesis that both receptor variants positively correlate with disease progression (Fig. 1a, b). Interestingly, AML relapsing patients were characterized by differential expression of P2X7RA and P2X7RB. Indeed, while P2X7RA expression was significantly reduced at relapse (Fig. 1c), P2X7RB mRNA substantially increased in this subset of patients (Fig. 1d) suggesting that chemotherapy would cause a decrease in P2X7RA expression and on the contrary a positive selection of P2X7RB in subjects refractory to treatment. In remitting AML patients, both P2X7RA and B expression was significantly decreased as compared to de novo diagnosed patients (Fig. 1c, d). These data suggest that individuals expressing high levels of P2X7RB might be resistant to chemotherapy and prone to relapse.

### DNR toxicity is increased by P2X7RA and reduced by P2X7RB expression

**Fig. 2:**
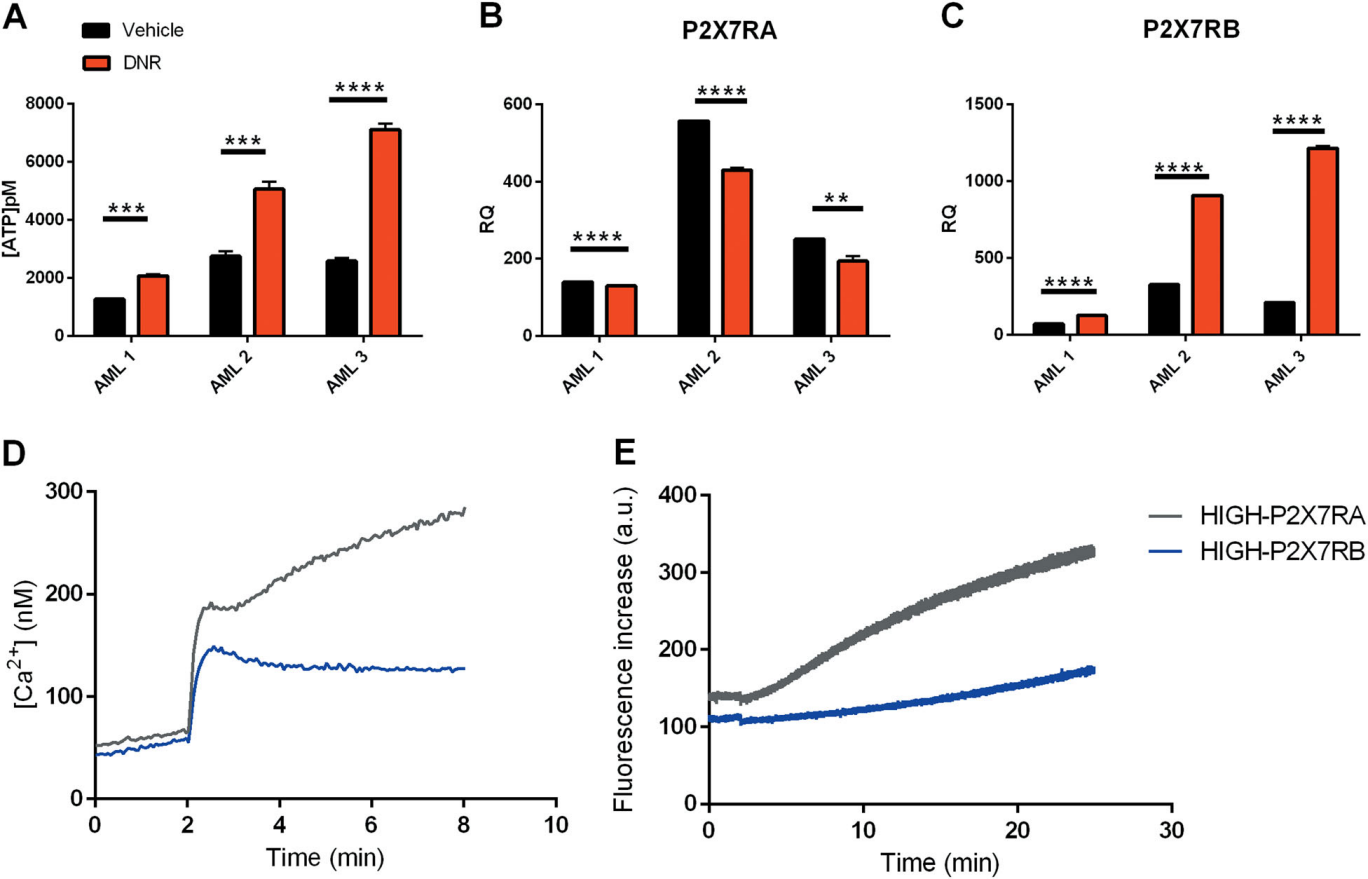
AML Blasts express functional P2X7RA and P2X7RB. **a** Extracellular ATP (pM) was measured in the culture supernatants as described in materials subjects and methods. AML blasts were plated and treated for 6 h with vehicle (PBS, black) and DNR (200 nM, red). mRNA expression of **b** P2X7RA and **c** P2X7RB in three representative AML patient blasts treated with vehicle (PBS, black) and DNR (200 nM, red). **d** Representative traces showing an increase of intracellular calcium following stimulation with 500 µM BzATP of HIGH-P2X7RA AML patient blasts (gray) and HIGH-P2X7RB AML patient blasts (blue). **e** Representative traces showing ethidium bromide uptake following stimulation with 500 µM BzATP of HIGH-P2X7RA AML patient blasts (gray) and HIGH-P2X7RB AML patient blasts (blue). Data are represented as mean ± SEM. *P ≤ 0.05; **P ≤ 0.01; ***P ≤ 0.001; ****P ≤ 0.0001.

**Fig. 3:**
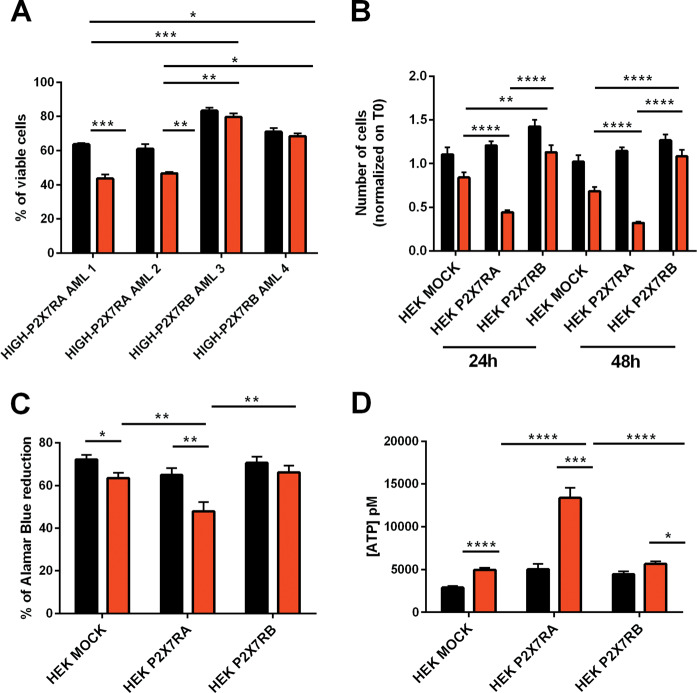
DNR toxicity is increased by P2X7RA and reduced by P2X7RB expression. **a** The panel represents the percentage of viable AML blasts characterized by HIGH-P2X7RA expression (AML 1 and AML 2) and HIGH-P2X7RB expression (AML 3 and AML 4) after 6 h of treatment with either vehicle (PBS, black) or DNR (200 nM, red). **b**, **c** HEK MOCK, HEK P2X7RA, and HEK P2X7RB were seeded at a concentration of 2 × 10^5^ and treated with vehicle (PBS, black) or DNR (200 nM, red) for 48 h. **b** The panel represents fold increase normalized on time 0 in cell numbers at 24 and 48 h after treatment. Data are presented as mean ± SEM. *n* = 26 microscopic fields for each group. Treatment with DNR causes a significant decrease in cell numbers for all cell types at both time points except for HEK P2X7RB at 24 h versus HEK P2X7RB at 48 h where the numbers of cells are not statistically different. These data are not depicted in the figure to increase clarity. Reported significance is relative to the comparison among different cell types all treated with DNR **P ≤ 0.01; ****P ≤ 0.0001. **c** Cell viability was assessed also by Alamar Blue assay, see “Materials, subjects, and methods.” 20,000 cell/100 µl were seeded in a 96-well plate and treated with vehicle (PBS, black) or DNR (200 nM, red) for 24 h. *n* = 18 for each group. Data are represented as mean ± SEM. *P ≤ 0.05; **P ≤ 0.01. **d** Extracellular ATP (pM) was measured in the culture supernatants as described in “Materials, subjects, and methods.” HEK MOCK, HEK P2X7RA, and HEK P2X7RB cells were treated for 24 h with vehicle (PBS black) or DNR (200 nM red). Data are represented as mean ± SEM. *n* = 6 for each group, *P ≤ 0.05; ***P ≤ 0.001,****P ≤ 0.0001.

All of the relapsing and remitting patients tested underwent first-line therapy including treatment with anthracyclines (DNR and/or idarubicin), this class of drugs is known to cause an increase of ATP in the TME^11,34^. Accordingly, DNR treatment caused the release of ATP from AML blasts (Fig. 2a) and, interestingly, caused downregulation of P2X7RA and upregulation of P2X7RB as seen in AML relapsing patients (Fig. 2b, c). When analyzing P2X7R activity as calcium channel or macropore we could clearly distinguish de novo patients expressing high levels of P2X7RA mRNA (HIGH-P2X7RA), which were characterized by high intracellular calcium and ethidium uptake (Fig. 2d, e) from those expressing high levels of P2X7RB (HIGH-P2X7RB) that only showed ion channel activity (Fig. 2d, e). Interestingly, HIGH-P2X7RB AML blasts when in colture were more vital and if treated with DNR less prone to cell death than HIGH-P2X7RA blasts (Fig. 3a). To further dissect the different roles played by P2X7RA versus P2X7RB in response to DNR we analyzed the effect of this drug on HEK-293 cells separately expressing P2X7RA, P2X7RB, or an empty vector (HEK P2X7RA, HEK P2X7RB, HEK MOCK)^23^. Interestingly, DNR toxicity was strongly increased in HEK P2X7RA cells as compared to mock controls, while expression of P2X7RB not only did not affect viability but even protected the cells from DNR-dependent death (Fig. 3b, c). These data were reproducible by both cell counts (Fig. 3b) and vitality assays with the Alamar Blue reagent (Fig. 3c). DNR treatment induced an ATP release from all tested cell lines (Fig. 3d) with a stronger effect on HEK P2X7RA, possibly due to increased cell death and consequent ATP release.

### P2X7RA facilitates cellular uptake of DNR

**Fig. 4:**
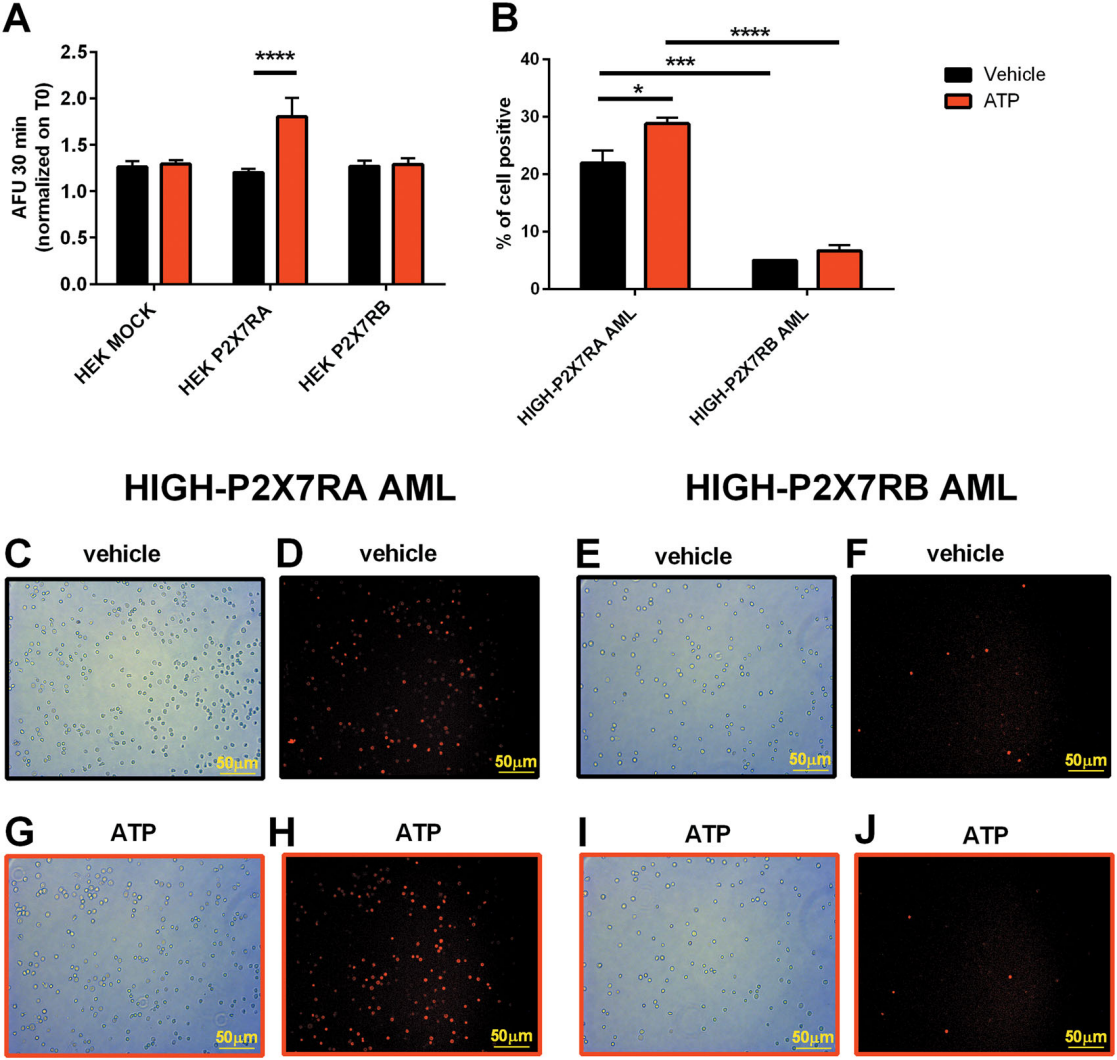
P2X7RA favors DNR uptake. HEK MOCK, HEK P2X7RA, and HEK P2X7RB cells (**a**) and AML patients blast (**b**–**j**) were incubated with DNR 1 µg/ml. Microscope captured fluorescence (545 nm) was emitted by DNR itself following excitation at 328 nm. **a** Images were acquired every 30 s for a total of 30 min after DNR application. Intracellular content of DNR was measured by the analysis of single-cell fluorescence. The figure depicts intracellular fluorescence fold increase, on time 0, 30 min after the application of vehicle (black) or 3 mM ATP (red). Data were obtained in three independent experiments. HEK mock vehicle *n* = 43, HEK mock ATP *n* = 70, HEK P2X7RA vehicle *n* = 88, HEK P2X7RA ATP *n* = 88, HEK P2X7RB vehicle *n* = 91, and HEK P2X7RB ATP *n* = 98. Data are represented as mean ± SEM. ****P ≤ 0.0001. **b** Images were acquired for a total of 15 min after DNR application. Intracellular content of DNR was measured by the analysis of single-cell fluorescence. The figure depicts the percentage of positive fluorescence cells on total cells, 15 min after the application of vehicle (black) or 3 mM ATP (red). Representative picture of respective bright field and fluorescence vehicle in HIGH-P2X7RA (**c**, **d**) and HIGH-P2X7RB (**e**, **f**) AML blasts. Representative picture of respective bright field and fluorescence ATP treatment in HIGH-P2X7RA (**g**, **h**) and HIGH-P2X7RB (**i**, **j**) AML blasts.

P2X7RA-mediated pore opening is known to mediate cell permeabilization to large molecules^14^, therefore we tested whether P2X7RA can further facilitate DNR-dependent cell death by favoring its cellular uptake. To test this hypothesis we took advantage of the natural red fluorescence emitted by DNR to measure its loading inside cells treated with 3 mM ATP (Fig. 4). As expected the only isoform able to promote DNR entry, possibly via macropore, was P2X7RA that significantly increased DNR uptake as compared to both control and P2X7RB expressing HEK cells (Fig. 4a). Similar results were obtained in HIGH-P2X7RA AML blasts, which, when treated with ATP, uploaded higher amounts of DNR than HIGH-P2X7RB patients cells (Fig. 4b–j).

### DNR and the P2X7R antagonist AZ10606120 when co-administered are more efficacious than separately in reducing leukemic growth in an in vivo xenograft model

**Fig. 5:**
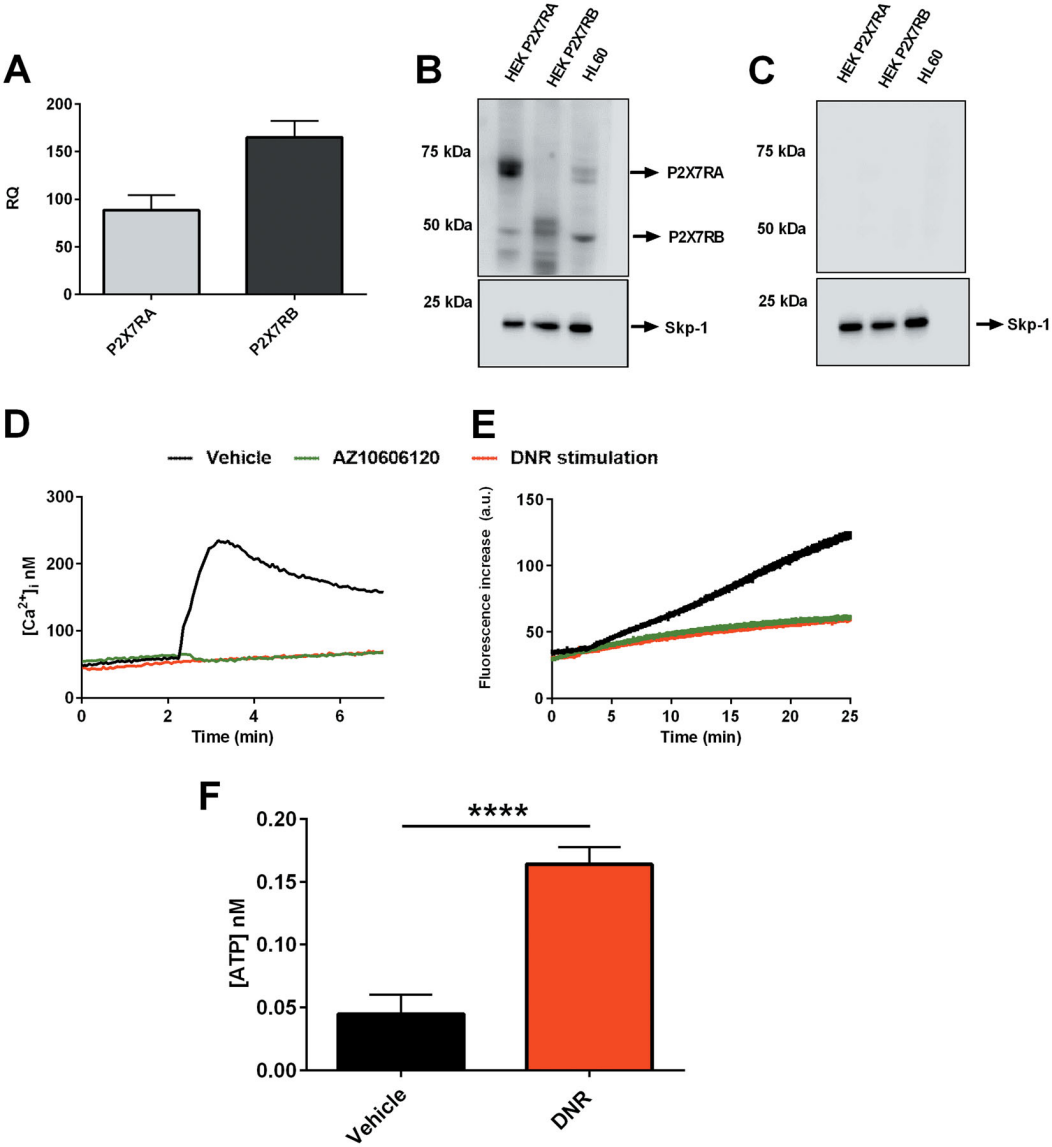
HL-60 human promyelocytic cell line expresses P2X7RA and B isoforms and releases ATP upon DNR treatment. **a** mRNA expression of P2X7RA (light gray) and P2X7RB (dark gray) in HL-60 cells (*n* = 6). **b** P2X7R isoforms expression revealed with an antibody directed against P2X7R extracellular domain, P2X7RA corresponds to a protein of ~70 KDa, while P2X7RB corresponds to a protein of ~50 KDa. Lane 1: HEK-293 transfected with P2X7RA, lane 2: HEK-293 transfected with P2X7RB, and lane 3: HL-60. Gel loading control Skp-1 (~20 kDa). **c** Immunoblot obtained with extracellular-domain directed anti-P2X7R antibody preincubated with blocking peptide (1/2 ratio, see “Materials, subjects, and methods”). Lane 1: HEK-293 transfected with P2X7RA, lane 2: HEK-293 transfected with P2X7RB, and lane 3: HL-60. Gel loading control Skp-1**. d** Representative traces showing an increment of intracellular calcium following stimulation with 500 µM BzATP alone (black) or applied after 5 min pretreatment with AZ10606120 (green) or following stimulation with 200 nM DNR alone (red). **e** Representative traces showing ethidium bromide uptake following stimulation with 500 µM BzATP alone (black) or applied after 5 min pretreatment with AZ10606120 (green) or following stimulation with 200 nM DNR alone (red). **f** Extracellular ATP (nM) was measured in the culture supernatants as described in “Materials, subjects, and methods.” HL-60 cells were plated at 5 × 10^5^ cells per ml and treated for 24 h with vehicle (PBS, black) and DNR (200 nM, red) (*n* = 8 for each group, vehicle versus DNR ****P < 0.0001).

**Fig. 6:**
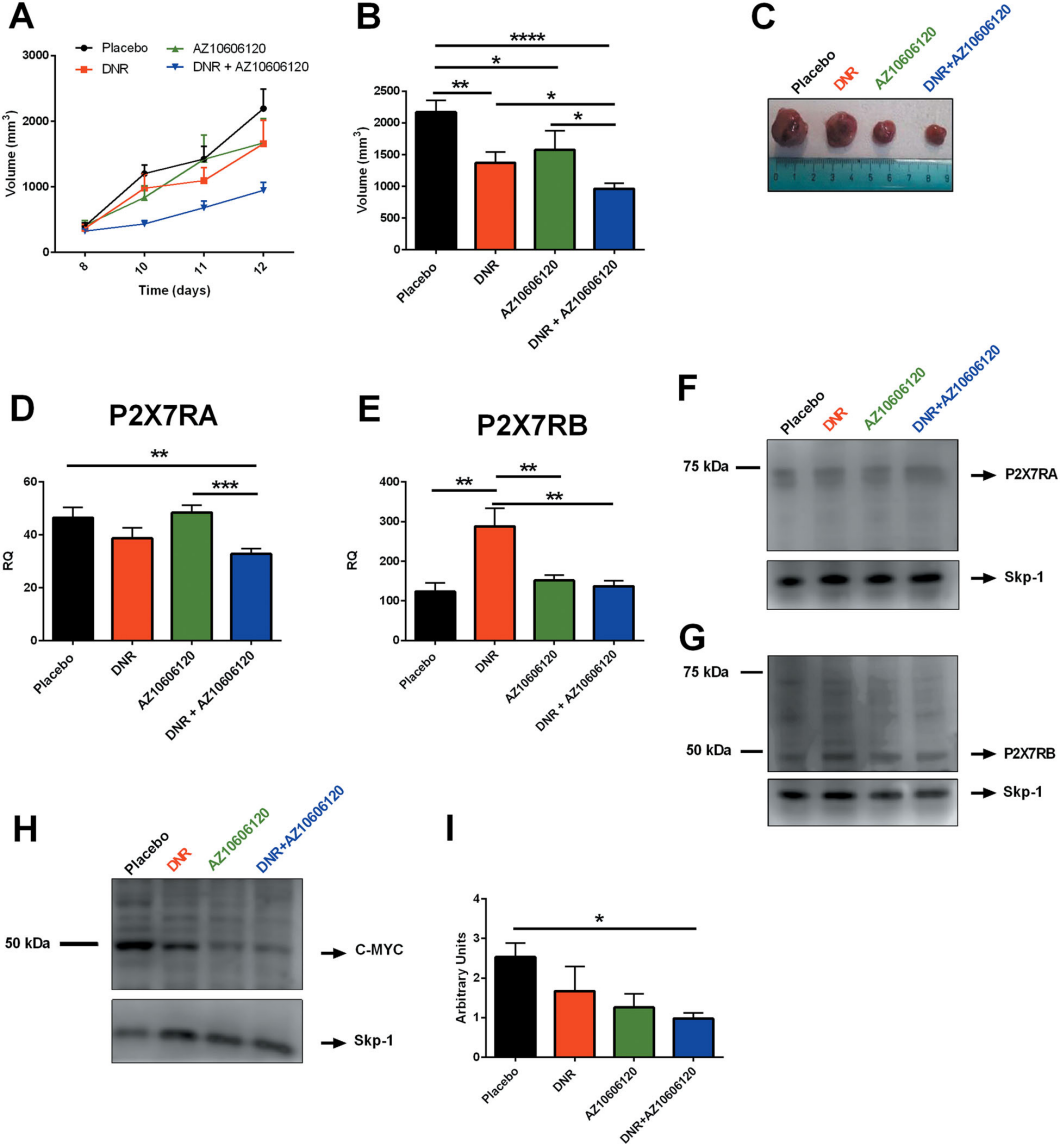
DNR and AZ10606120 combined treatment is more effective in reducing HL-60 growth in nude mice in comparison to their single administration, affecting P2X7RA, P2X7RB, and c-myc expression. **a**–**i** Female nude mice were subcutaneously inoculated into the fat of the right limb with 5 × 10^6^ HL-60 cells. DNR (0.75 mg/kg), AZ10606120 (5 mg/kg), and both drugs combined were i.m. administered to mice at post-inoculum days 8 and 10. **a**, **b** Placebo depicted in black, DNR depicted in red, AZ10606120 depicted in green, DNR + AZ10606120 depicted in blue, *n* = 12 for each group. **a** At the indicated time points, tumor volume was in vivo assessed by a caliper. Data are represented as mean ± SEM. Significance is not depicted in the figure to increase clarity. Placebo versus DNR + AZ10606120 at day 10 *P* ≤ 0.0001, at day 11 *P* ≤ 0.01, at day 12 *P* ≤ 0.001. DNR versus DNR + AZ10606120 at day 10 *P* ≤ 0.05. AZ10606120 versus DNR + AZ10606120 at day 10 *P* ≤ 0.01. **b** Ex vivo tumor volume assessed at day 12 by a caliper. Data are represented as mean ± SEM. *P ≤ 0.05; **P ≤ 0.01; ****P ≤ 0.0001. **c** Representative picture of tumors from treated mice at post-inoculum day 12. **d**, **e** P2X7R isoforms A and B mRNA tumor levels were evaluated by real-time PCR as described in “Materials, subjects, and methods.” **d** P2X7RA mRNA expression. Data are represented as mean ± SEM. **P ≤ 0.01; ***P ≤ 0.001. **e** P2X7RB mRNA expression. Data are represented as mean ± SEM. **P ≤ 0.01. **f** P2X7RA protein (~70 kDa) expression in excised tumors was evaluated by western blot with an antibody directed against P2X7R C terminal domain. Gel loading control Skp-1 (~20 kDa). **g** P2X7RB protein (~50 kDa) expression in excised tumors was evaluated by western blot with an antibody directed against P2X7R extracellular domain. Gel loading control Skp-1 (~20 kDa). **h** Representative Immunoblot showing c-myc expression (~50 KDa) in excised tumors. Gel loading control Skp-1 (~20 kDa). **i** Densitometric analysis of c-myc expression in HL-60 derived tumors normalized on Skp-1 loading control. Data were obtained in three independent experiments and are relative to three different tumor masses per group. Data are represented as mean ± SEM. *P ≤ 0.05.001.

To analyze the role of P2X7R isoforms in an AML in vivo model, we took advantage of HL-60 human promyelocytic cell line that expresses both P2X7RA and B at the mRNA and protein level (Fig. 5a, b), shows functional activity of the receptor as an ion channel and macropore (Fig. 5c, d), and releases ATP upon DNR treatment (Fig. 5e). We subcutaneously injected HL-60 cells in athymic nude mice. Tumor-bearing animals were then treated with intramass injections of DNR, the P2X7R antagonist AZ10606120, or both compounds together. Both drugs significantly reduced experimental leukemia growth (Fig. 6a–c). Moreover, coadministration proved to be more efficacious than single-agent treatment (Fig. 6a–c). DNR effect on P2X7R isoforms expression in HL-60 derived tumors was similar to patients’ data as P2X7RB expression was significantly increased (Fig. 6e), while P2X7RA showed a tendency to reduction (Fig. 6d). Interestingly, coadministration of DNR with AZ10606120 reduced both P2X7RA and P2X7RB (Fig. 6d–g). We had previously demonstrated that P2X7R antagonism reduced N-myc levels in neuroblastoma murine models^35^, therefore we asked whether AZ10606120 could similarly affect the expression of c-myc that is a well-known oncogene in AML and MDS^36^. Figure 6h, i shows a reduction of c-myc expression associated with P2X7R blockade in HL-60 xenografts. Interestingly, while DNR did not affect c-myc expression, P2X7R blockade alone tended to reduce and AZ10606120 in combination with DNR strongly diminished c-myc levels.

## Discussion

The P2X7R was previously reported to be expressed and functional in AML^37^ and its targeting reduced leukemic growth in AML experimental models^12^. In vitro experiments also showed that high doses of ATP selectively disrupted the growth of leukemic blasts versus normal haemopoietic stem cells^10^. However, these studies focused on P2X7RA isoform that is known to mediate both cell proliferation upon stimulation by low doses of agonist and cell death through the opening of a large unselective pore in the presence of millimolar concentrations of ATP^38^. Humans also express another splice variant of the receptor named P2X7RB^22^, which while retaining the proliferation-promoting activity loses the ability to mediate the opening of the cytotoxic pore^23^. Limited evidence is available on the physio-pathological role of P2X7RB, we know that it is expressed mainly in the central and peripheral nervous system and in leukemic cells^23^ and that, its expression associates to stem cell differentiation^39^. In solid cancers, P2X7RB was hypothesized to participate in proliferation^24^, matrix invasion,^26^ and tumor spreading^25^. To better understand the different roles of P2X7RA and P2X7RB splice variants in leukemias, we investigated the expression of these isoforms in patients affected by MDS or AML and in AML relapsing and remitting patients. Both P2X7RA and P2X7RB mRNA levels were strongly increased in firstly diagnosed AML as compared to MDS, supporting the hypothesis that both receptor variants positively correlate with disease progression. Interestingly, AML relapsing patients after chemotherapy were characterized by differential expression of P2X7RA and P2X7RB as compared to de novo patients as while P2X7RA expression was significantly reduced, P2X7RB expression significantly increased. On the contrary, in remitting AML patients both P2X7RA and B expression was significantly decreased. These data suggest that chemotherapy may cause a decrease in P2X7RA expression while positively modulating P2X7RB and therefore that individuals expressing high levels of P2X7RB could be resistant to chemotherapy, prone to relapse and refractory to treatment.

**Fig. 7:**
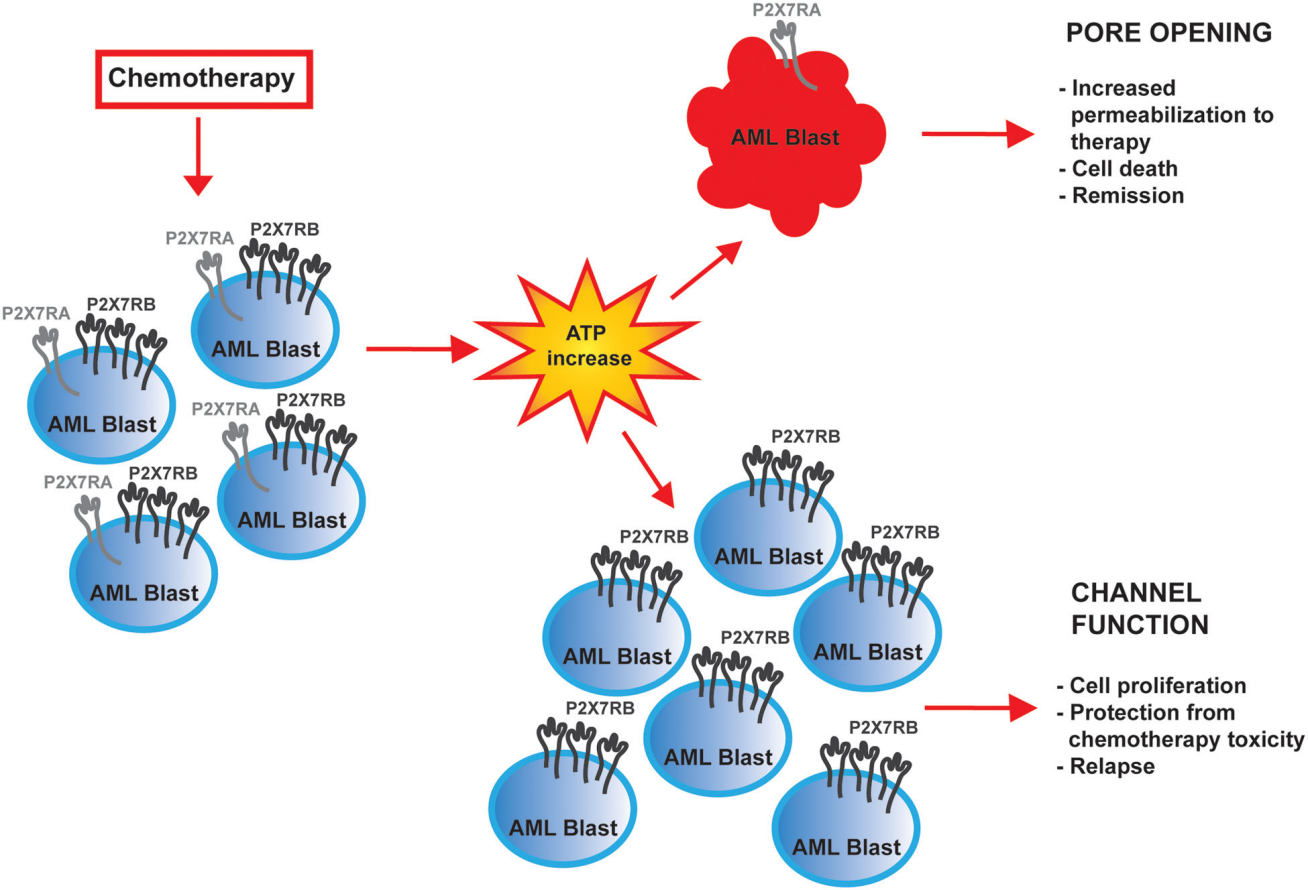
Chemotherapy with anthracyclines induces ATP release in the tumor microenvironment. P2X7RA expressed by AML blasts in the presence of high ATP concentration mediates the opening of a large nonselective pore facilitating intracellular uptake of anticancer drugs that induce cell death. On the other hand, the increase of ATP allows the proliferation of P2X7RB expressing blasts, unable to form the cytotoxic pore but still able to activate the channel function of the receptor that protect cells from chemotherapy-dependent death and favors the relapse of AML.

We recently demonstrated that the chemotherapeutic DNR can cause an increase of ATP in the leukemic microenvironment^11^ and confirmed these data in all cell types tested in the present study. Based on these findings, we hypothesized that chemotherapy could cause a release of ATP from dying leukemic cells, which will, on one hand, kill P2X7RA overexpressing blasts through macropore formation and, on the other facilitate the proliferation of P2X7RB expressing blasts that do not undergo cell death but on the contrary exploit ATP-dependent proliferation (see Fig. 7). This hypothesis was confirmed by the finding that DNR toxicity was increased in HIGH-P2X7RA AML blasts, while the overexpression of P2X7RB protected these cells from DNR-dependent death, and further supported by similar data obtained in HEK-293 cells separately transfected with either P2X7RA or P2X7RB. These findings are in line with a differential role of the two isoforms in response to chemotherapy (see Fig. 7) and with P2X7RB upregulation in AML relapsing patients. To further dissect the mechanisms underlying P2X7RA facilitation of DNR toxicity we asked whether P2X7RA-mediated pore opening could facilitate DNR uptake by tumor cells, as previously demonstrated for the uptake of its analog doxorubicin in macrophages^17^. Indeed, this proved to be the case as DNR uptake by both HEK P2X7RA and HIGH-P2X7RA AML blasts stimulated with 3 mM ATP was higher than in HEK mock controls, HEK P2X7RB cells, and HIGH-P2X7RB AML blasts, respectively.

To validate patients’ data in an in vivo model of leukemia we took advantage of HL-60 human promyelocytic cell line, which expresses both P2X7R isoforms, can form the cytolytic pore and releases ATP upon DNR stimulation. HL-60-derived leukemia-bearing mice were treated with DNR and with a P2X7R antagonist, alone or in coadministration. Both compounds significantly reduced leukemia growth, but their combination was more efficient than single-drug treatment. DNR effect on P2X7R isoforms expression was similar to what was seen in relapsing patients as P2X7RB expression was significantly increased by DNR, while P2X7RA showed a tendency to decrease. Interestingly, coadministration of DNR with P2X7R antagonist normalized both P2X7RA and B levels and reduced intratumor levels of the c-myc oncogene. These data are in line with our previous demonstration that P2X7R blockade decreases expression of the N-myc oncogene, another member of the myc family^35^, and suggest a mechanism for P2X7R-mediated transformation in AML. c-myc is commonly overexpressed in both AML and MDS^36,40^ as it is a central transcription factor in the maturation of hematopoietic stem cells, thus exerting a critical function in hematopoietic malignancies^41^. Moreover, c-myc is involved in AML cells resistance to chemotherapy and therefore identifying new drugs able to downmodulate c-myc levels might greatly improve the therapeutic approach of AML relapsing patients^42,43^. AML relapse is the main cause of patients’ death and efforts to improve their survival is an important goal for onco-hematologists to ensure patients’ recovery. Our study suggests that P2X7R isoforms could be exploited as valuable prognostic markers to predict response to chemotherapy. Moreover, P2X7R could be an interesting target for a new combined therapy in AML. Based on our data, we suggest a therapeutic approach with DNR administered as first-line, to eliminate P2X7RA-expressing leukemic cells. P2X7R antagonists could be administered in a second phase to those that developed chemotherapy resistance and whose blasts are positive for P2X7RB expression. Of interest, several P2X7R targeting drugs are under clinical trials for other pathologies and proved to be well tolerated with limited side effects thus favoring their use also in oncologic patients^5,44,45^.

## Supplementary information

supplemental table 1

supplementary table legend

supplemental figure 1

supplementary figure legend
