# Supplementary material for: Differential sensitivity of acute myeloid leukemia cells to daunorubicin depends on P2X7A versus P2X7B receptor expression
Source: Cell Death Dis. 2020 Oct 18;11(10):876. doi: 10.1038/s41419-020-03058-9 (PMC7569086; doi:10.1038/s41419-020-03058-9)
Supplement: Supplementary file 1 — supplemental table 1 [file 41419_2020_3058_MOESM1_ESM.docx]

| # Patient | Phase | Age | Sex | Fab | Karyotype | Genotype | Risk |
| --- | --- | --- | --- | --- | --- | --- | --- |
| AML 1 | ONSET | 46 | F | M1 | t(6;9)(p23;q34) | FLT3-ITD+,NPM1wt | high |
| AML 2 | ONSET | 63 | M | M4-M5 | normal | NPM1mut, FLT3mut | intermediate |
| AML 3 | ONSET | 55 | F | M4 | inv(16)[19] | FLT3wt, NPM1wt, DNMT3Awt | low |
| AML 4 | ONSET | 66 | F | M1-M2 | normal | NPM1mut, FLT3wt | low |
| AML 5 | ONSET | 48 | M | M1 | complex | FLT3wt, NPMwt, DNMT3Awt, TP53wt | high |
| AML 6 | ONSET | 59 | F | NA | normal | NA | high |
| AML 7 | ONSET | 68 | M | M2-M4 | inv(16) | NA | low |
| AML 8 | ONSET | 39 | M | M3 | t(15;17)(q22;q12) | PML/RARα, NPM1wt, FLT3wt | - |
| AML 9 | ONSET | 63 | F | M4 | normal | NPM1mut, FLT3wt | low |
| AML 10 | ONSET | 64 | F | M1 | normal | FLT3mut, NPM1mut, DNMT3Awt, TP53wt | high |
| AML 11 | ONSET | 68 | F | M2-M4 | normal | FLT3-ITD+, FLT3-TKDwt, NPM1mut, DNMT3Amut | intemediate |
| AML 12 | ONSET | 46 | F | M2 | t(8;21) | NA | low |
| AML 13 | ONSET | 54 | M | NA | t(8;21) | NPM1wt, FLT3wt | low |
| AML 14 | ONSET | 64 | M | M0-M1 | normal | FLT3-ITDwt, NPM1mut | low |
| AML 15 | ONSET | 75 | M | NA | NA | NPM1mut, FLT3wt | low |
| AML 16 | ONSET | 69 | M | M3 | t(15;17) | PML-RARa BCR1, NPM1mut, FLT3wt | - |
| AML 17 | ONSET | 77 | F | M3 | t(15;17) | PML-RARα | - |
| AML 18 | ONSET | 90 | M | NA | NA | NA | NA |
| AML 19 | ONSET | 68 | M | M0-M1 | NA | NPM1wt, FLT3wt | intemediate |
| AML 20 | ONSET | 17 | M | M5 | 47, +der(3) del(3), t(10,11,19) | NPM1wt, FLT3wt | high |
| AML 21 | ONSET | 60 | F | M5 | complex | NPM1wt , FLT3 wt, TP53+ , DNMT3Awt | high |
| AML 22 | ONSET | 73 | M | M2 | normal | FLT3wt, NPM1mut, TP53wt | low |
| AML 23 | ONSET | 70 | M | M2 | NA | FLT3-ITD+, NPM1mut, TP53 wt | high |
| AML 24 | ONSET | 78 | M | M0 | NA | NPM1wt, FLT3mut | high |
| AML 25 | ONSET | 51 | F | M5 | normal | NPM1mut, FLT3mut | intemediate |
| AML 26 | ONSET | 46 | F | M4 | t(16;6) | NPM1wt, FLT3wt,TP53wt | low |
| AML 27 | ONSET | 19 | M | NA | 46xx t(6:11) | NPM1wt, FLT3wt | high |
| AML 28 | ONSET | 21 | M | M0-M1 | complex | FLT3wt, NPM1wt, DNMT3Awt | high |
| AML 29 | ONSET | 60 | F | M5 | t(4) | FLT3-TKD+, NPM1mut, DNMT3Amut | low |
| AML 30 | ONSET | 38 | M | NA | t(6;14)(q25;q32), t(1;5)(p22;q33) | FLT3wt; IDH1/IDH2wt; NPM1wt | high |
| AML 31 | ONSET | 69 | M | NA | normal | FLT3-ITD+, FLT3 TKDwt, TP53wt, IDH1/IDH2wt, DNMT3Amut | high |
| AML 32 | ONSET | 72 | M | NA | normal | NA | intermediate |
| AML 33 | ONSET | 62 | M | NA | t(9:22) | FLT3wt, DNMT3Awt, BCR-ABL+ | high |
| AML 34 | ONSET | 69 | F | M4 | 46xx, t(X;9)(p11;p13) | NA | intermediate |
| AML 35 | ONSET | 26 | M | M2 | 46,XY,inv(16)(p13q22)(18)/47,XY,+22,inv(16)(p13q22)(2) | NPM1wt, TP53wt, FLT3wt | low |
| AML 36 | ONSET | 65 | M | NA | normal | FLT3-ITD+ | high |
| AML 37 | ONSET | 61 | F | NA | normal | FLT3-ITD+ | high |
| AML 38 | ONSET | 72 | F | NA | -7; t(11;19); t (3;5) | FLT3wt, TP53 wt NPM1wt | high |
| AML 39 | ONSET | 72 | F | NA | 47,XX,+X(1)/47,XX,+X,add(7)(q34)(19) | NPM1wt, FLT3wt | intemediate |
| AML 40 | ONSET | 79 | M | NA | normal | NA | intermediate |
| AML 41 | ONSET | 73 | F | M5 | normal | NPM1mut, FLT3 ITD+ | intermediate |
| AML 42 | ONSET | 67 | M | NA | normal | NPM1wt, FLT3mut | high |
| AML 43 | ONSET | 42 | M | M5 | NA | FLT3mut, NPM1mut | intermediate |
| AML 44 | ONSET | 79 | M | M2 | NA | NA | NA |
| AML 45 | ONSET | 71 | M | NA | 96 xxyy add 5q del 4p | FLT3wt, IDH1/2wt, TP53wt | high |
| AML 46 | ONSET | 55 | F | M0-M1 | 46,XX,der(4)t(1;4)(q32;q31) | NPM1wt, TP53wt, FLT3wt | intermediate |
| AML 47 | ONSET | 31 | M | M0 | normal | FLT-ITD+, NPM1mut | intermediate |
| AMLREL1 | RELAPSE | 52 | F | NA | NA | NA | NA |
| AMLREL2 | RELAPSE | 53 | F | M3 | t(15,17) | NA | NA |
| AMLREL3 | RELAPSE | 42 | F | M4 | normal | FLT3-ITD+, NPM1mut | intermediate |
| AMLREL4 | RELAPSE | 54 | M | M3 | t(15,17) | NA | NA |
| AMLREL5 | RELAPSE | 67 | M | NA | normal | FLT3wt | intermediate |
| AMLREL6 | RELAPSE | 66 | F | NA | NA | FLT3-ITD+ | high |
| AMLREM1 | REMISSION | 63 | F | - | NA | NA | NA |
| AMLREM2 | REMISSION | 19 | M | - | NA | NA | NA |
| AMLREM3 | REMISSION | 26 | M | - | NA | NA | NA |
| AMLREM4 | REMISSION | 72 | M | - | NA | NA | NA |
| AMLREM5 | REMISSION | 31 | M | - | NA | NA | NA |
| MDS1 | ONSET | 65 | M |  | 46,XY,del(13)(q12q14)[3]/46,XY[17] |  | high/very high |
| MDS2 | ONSET | 50 | F |  | 46,XX [20] |  | NA |
| MDS3 | ONSET | 78 | M |  | 46,XY [20] |  | NA |
| MDS4 | ONSET | 79 | F |  | 46,XX [20] |  | NA |
| MDS5 | ONSET | 62 | M |  | 46,XY [20] |  | int2/high |
| MDS6 | ONSET | 81 | F |  | 46,XX,del(20)(q11q13)[20] |  | low/low |
| MDS7 | ONSET | 86 | M |  | 46,XY [20] |  | low/low |
| MDS8 | ONSET | 89 | M |  | 46,XY [20] |  | low/low |
| MDS9 | ONSET | 77 | M |  | 46,XY [20] |  | NA |
| MDS10 | ONSET | 72 | M |  | 46,XY [20] |  | NA |
