# Supplementary material for: Differential sensitivity of acute myeloid leukemia cells to daunorubicin depends on P2X7A versus P2X7B receptor expression
Source: Cell Death Dis. 2020 Oct 18;11(10):876. doi: 10.1038/s41419-020-03058-9 (PMC7569086; doi:10.1038/s41419-020-03058-9)
Supplement: Supplementary file 2 — supplementary table legend [file 41419_2020_3058_MOESM2_ESM.docx]

**Supplementary Table 1.** The table summarizes principal AML and MDS patients’ characteristics including disease phase, age, sex, French-American-British (Fab) classification, karyotype and genotype abnormalities and attributed risk. No blasts from patients that were previously subject to bone marrow transplantation as therapeutic intervention were analyzed.
