# Supplementary figures and images for: Differential sensitivity of acute myeloid leukemia cells to daunorubicin depends on P2X7A versus P2X7B receptor expression
Source: Cell Death Dis. 2020 Oct 18;11(10):876. doi: 10.1038/s41419-020-03058-9 (PMC7569086; doi:10.1038/s41419-020-03058-9)

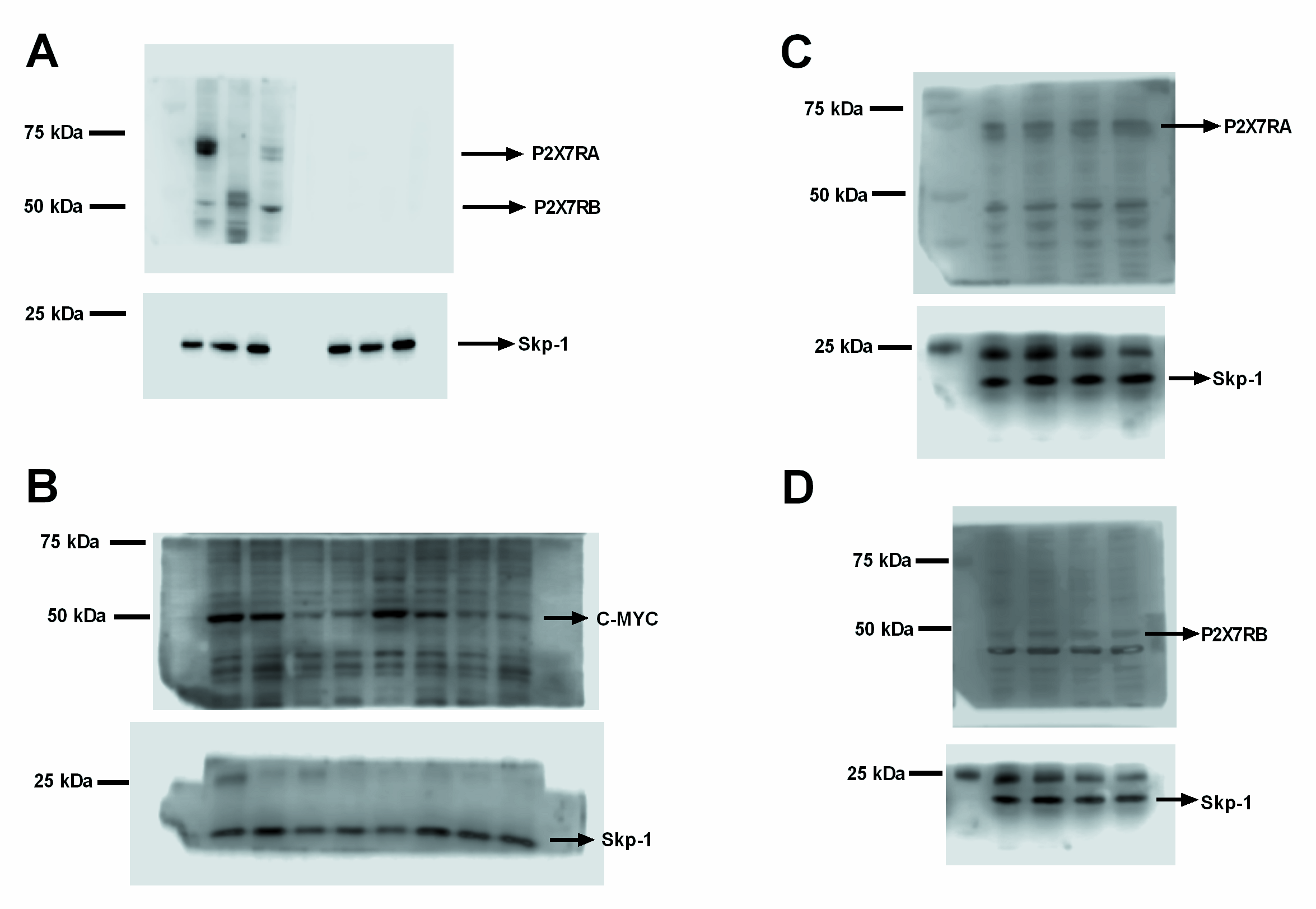

Supplement: Supplementary file 3 — supplemental figure 1 [file 41419_2020_3058_MOESM3_ESM.tif]
