# Supplementary material for: Differential sensitivity of acute myeloid leukemia cells to daunorubicin depends on P2X7A versus P2X7B receptor expression
Source: Cell Death Dis. 2020 Oct 18;11(10):876. doi: 10.1038/s41419-020-03058-9 (PMC7569086; doi:10.1038/s41419-020-03058-9)
Supplement: Supplementary file 4 — supplementary figure legend [file 41419_2020_3058_MOESM4_ESM.docx]

Supplementary Figure 1 legend:

Panel A; Untrimmed Western blot corresponding to panels B and C figure 5.

Panel B: Untrimmed Western blot corresponding to panel H figure 6. The lanes shown in figure 6 correspond to the last four.

Panel C: Untrimmed Western blot corresponding to panel F figure 6. All lanes are shown in figure 6.

Panel D Untrimmed Western blot corresponding to panel G figure 6. All lanes are shown in figure 6.
